# Supplementary material for: Voltage sensors of a Na+ channel dissociate from the pore domain and form inter-channel dimers in the resting state
Source: Nat Commun. 2023 Dec 19;14:7835. doi: 10.1038/s41467-023-43347-3 (PMC10730821; doi:10.1038/s41467-023-43347-3)
Supplement: Supplementary file 1 — Supplementary Information [file 41467_2023_43347_MOESM1_ESM.pdf]

Supplementary information

Voltage sensors of a Na^+ channel dissociate from the pore domain and form inter-channel dimers in the resting state

Ayumi Sumino[1,2]*, Takashi Sumikama[1]*, Mikihiro Shibata[1,2], Katsumasa Irie[3]*

1 Nano Life Science Institute (WPI-NanoLSI), Kanazawa University, Kanazawa, 920-1192, Japan
2 Institute for Frontier Science Initiative, Kanazawa University, Kanazawa, 920-1192, Japan
3 Department of Biophysical chemistry School of Pharmaceutical Science, Wakayama Medical University, Wakayama, 640-8156, Japan
* Corresponding authors
Correspondence: sumino@staff.kanazawa-u.ac.jp, sumi@staff.kanazawa-u.ac.jp, kirie@wakayama-med.ac.jp

Table of Contents

Supplementary Fig.1. HS-AFM image of the Na_vAb (KAV) cluster in a lipid bilayer.
Supplementary Fig.2. Probability of VSD detection around PDs.
Supplementary Fig.3. Distance between two particles derived from a single VSD.
Supplementary Fig.4. Simulated AFM image of a VSD.
Supplementary Fig.5. Estimation of the maximum distance from the center of the PD to the S1-S2 particle.
Supplementary Fig.6. Other examples of trajectories and eigenvectors of PCA for the WT.
Supplementary Fig.7. Other examples of trajectories and eigenvectors of PCA for the N49K mutant.
Supplementary Fig.8. Other examples of trajectories and eigenvectors of PCA for the E32Q/N49K mutant.
Supplementary Fig.9. Density dependence of the contact probability between particles mimicking resting Na_v channels.
Supplementary Fig.10. E32Q/N49K mutant without a lipid bilayer.

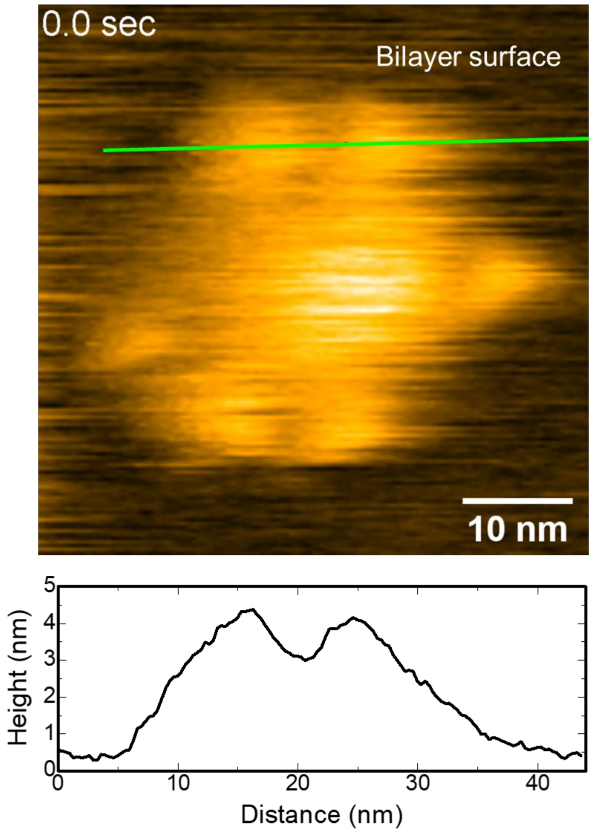

Supplementary Fig.1. HS-AFM image of the Na_vAb (KAV) cluster in a lipid bilayer.

The height profile along the green line is shown. Particles formed cluster in the bilayer and protruded approximately 3-4 nm from the lipid bilayer surface. The height is too high to consider that the molecule appropriately reconstituted in the lipid bilayer since other clearly observed Na_vAb channels showed only approximately 1 nm protrusion. The corresponding HS-AFM movie is Movie S1.

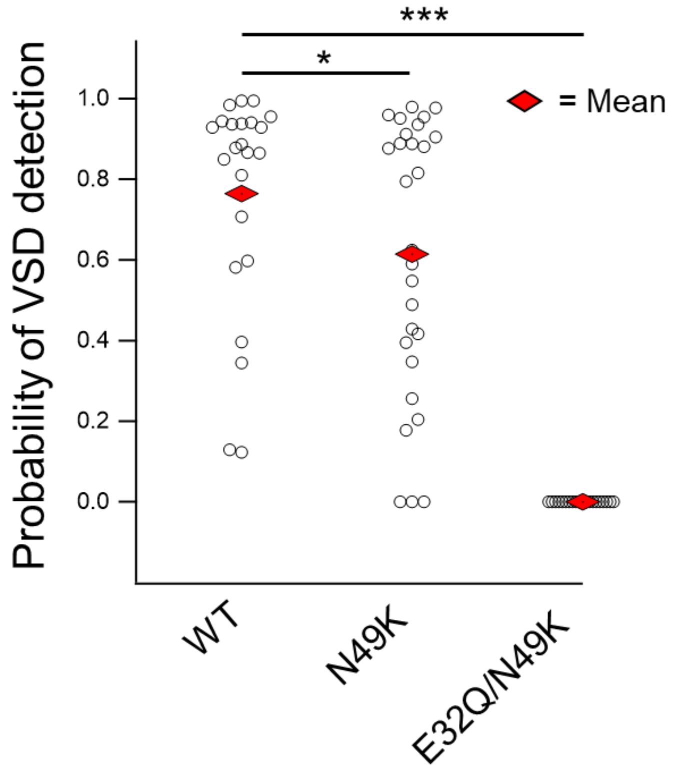

Supplementary Fig.2. Probability of VSD detection around PDs.

Probability of VSD detection = total frame number detecting VSD/total frame number of the HS-AFM movie. Number of channels; 5, 7, and 5 for the WT, N49K mutant and E32Q/N49K mutant, respectively. Since the Na_vAb channel is a homotetramer, a single channel yields four plots.

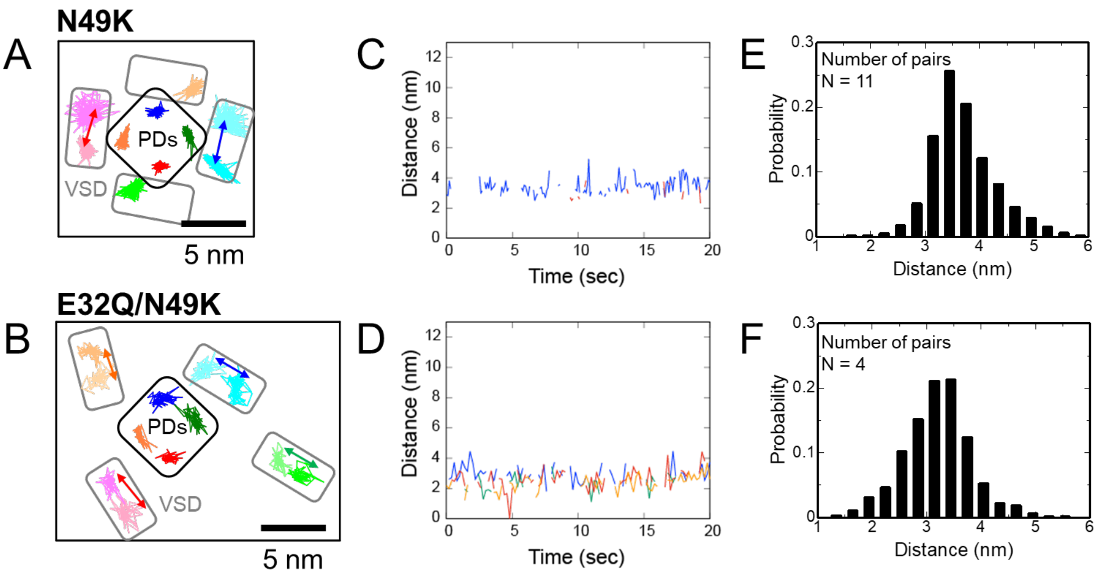

Supplementary Fig.3. Distance between two particles derived from a single VSD.

(A and B) Trajectories of Na_vAb channels. Trajectories surrounded by black squares and gray rectangles correspond to PDs and VSD, respectively. (C and D) Time series of the distance between two particles derived from the same VSD (S1-S2, S3-S4) indicated by arrows in A and B. (E and F) Histograms of the distance between two particles derived from the same VSD. The numbers of pairs (channels) are 11 (4) and 4 (1) for E and F, respectively. The total data numbers are 2416 and 644 for E and F, respectively. The analyzed constructs are the N49K mutant for (A, C and E) and the E32Q/N49K mutant for (B, D and F). Frame rates: 5 frame/sec for A, 10 frame/sec for B.

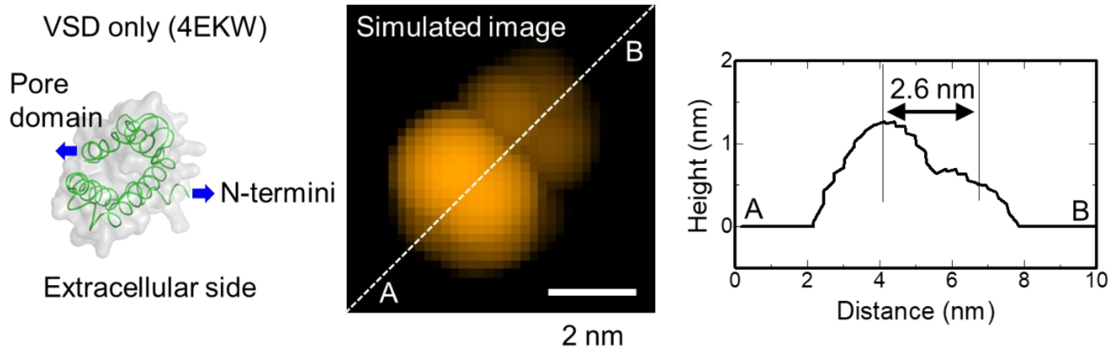

Supplementary Fig.4. Simulated AFM image of a VSD.

Simulated AFM image of the extracellular surface of the single VSD (4ekw), with the virtual membrane surface placed at a height of 4 nm from the intracellular end. The height profile along the dashed line is shown in the right panel.

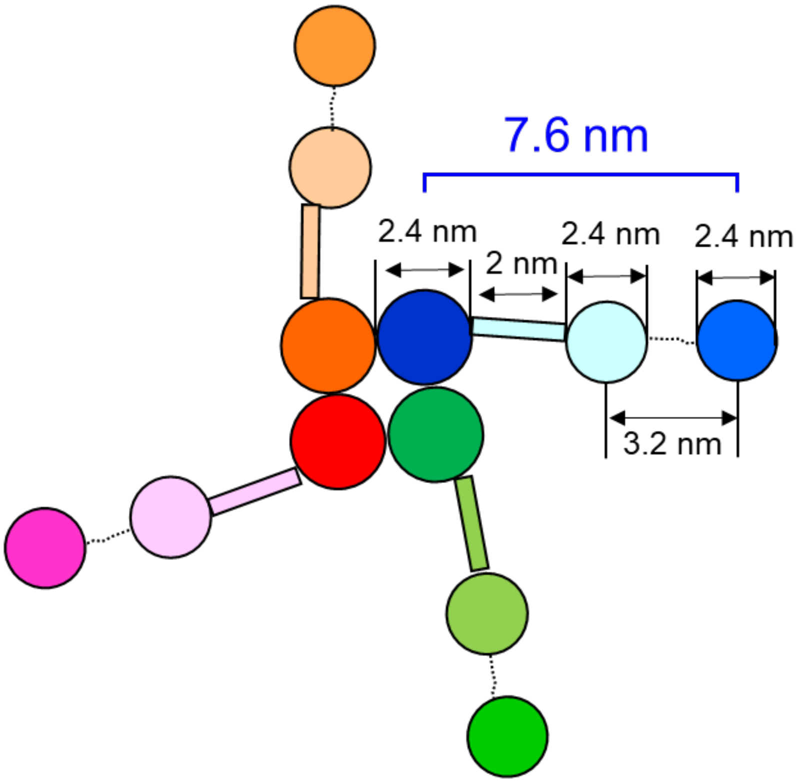

Supplementary Fig.5. Estimation of the maximum distance from the center of the PD to the S1-S2 particle.

The length of the S4-S5 linker is 2 nm when it forms an α-helix, the distance between the S1-S2 and S3-S4 particles is 3.2 nm as evidenced by the AFM images (Figure S4), and assuming the diameter of each particle is 2.4 nm (a single helix is 1.2 nm, so the long diameter is approximately 2.4 nm for two helices) and the S4-S5 linker is dissociated from the PDs, the maximum distance between PD and S1-S2 is approximately 7.6 nm

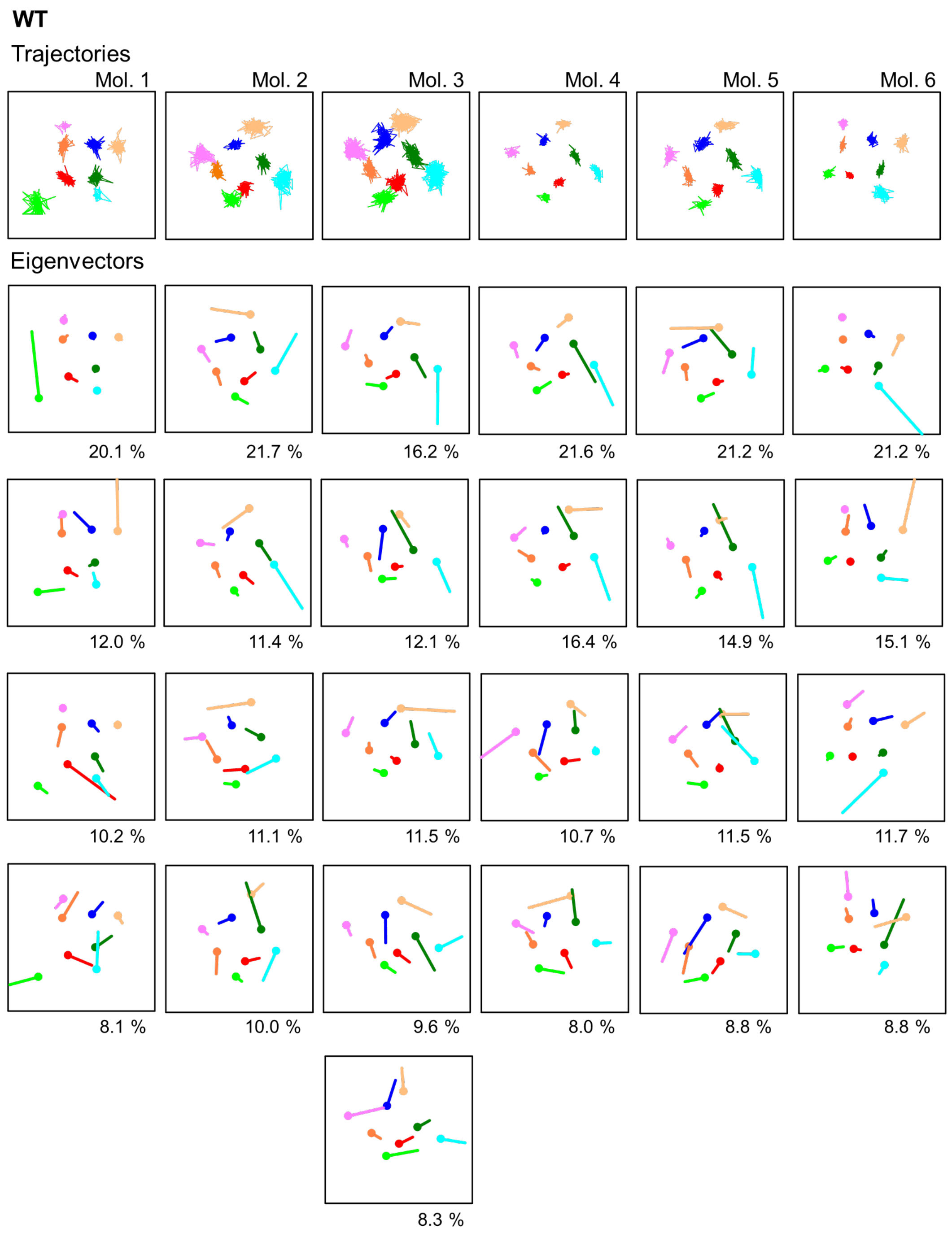

Supplementary Fig.6. Other examples of trajectories and eigenvectors of PCA for the WT. Trajectories (upper panels) and their eigenvectors of PCA (lower panels) are shown. Below the eigenvectors, the ratio of the eigenvalues of the relevant mode to the sum of the eigenvalues of all modes is shown as a percentage. Eigenvectors are shown in order of increasing components until the total is 50%. Plot area: 15 nm square.

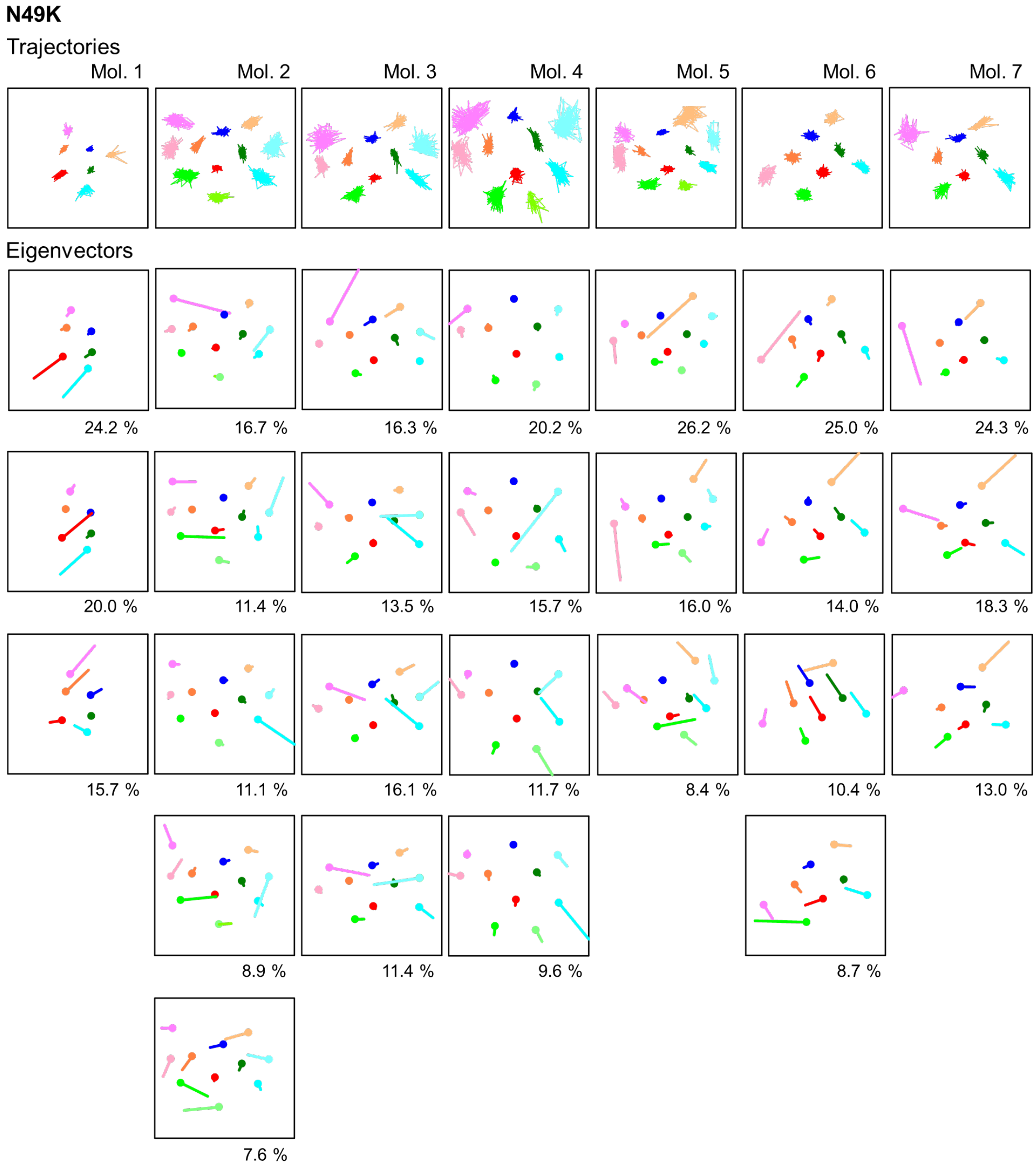

Supplementary Fig.7. Other examples of trajectories and eigenvectors of PCA for the N49K mutant.

Trajectories (upper panels) and their eigenvectors of PCA (lower panels) are shown. Below the eigenvectors, the ratio of the eigenvalues of the relevant mode to the sum of the eigenvalues of all modes is shown as a percentage. Eigenvectors are shown in order of increasing components until the total is 50%. Plot area: 15 nm square.

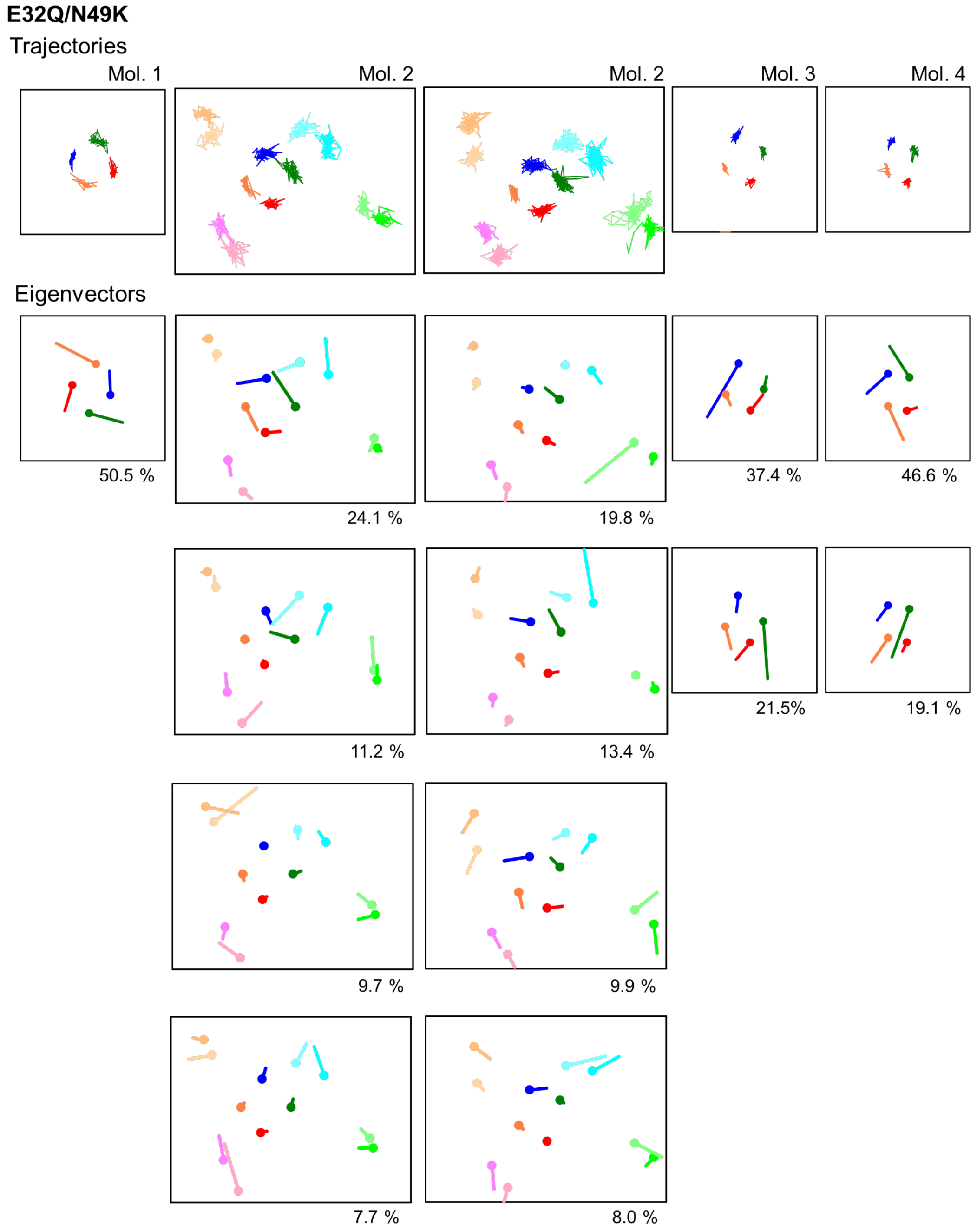

Supplementary Fig.8. Other examples of trajectories and eigenvectors of PCA for the E32Q/N49K mutant.

Trajectories (upper panels) and their eigenvectors of PCA (lower panels) are shown. Below the eigenvectors, the ratio of the eigenvalues of the relevant mode to the sum of the eigenvalues of all modes is shown as a percentage. Eigenvectors are shown in order of increasing components until the total is 50%. Plot area: 22×17 nm.

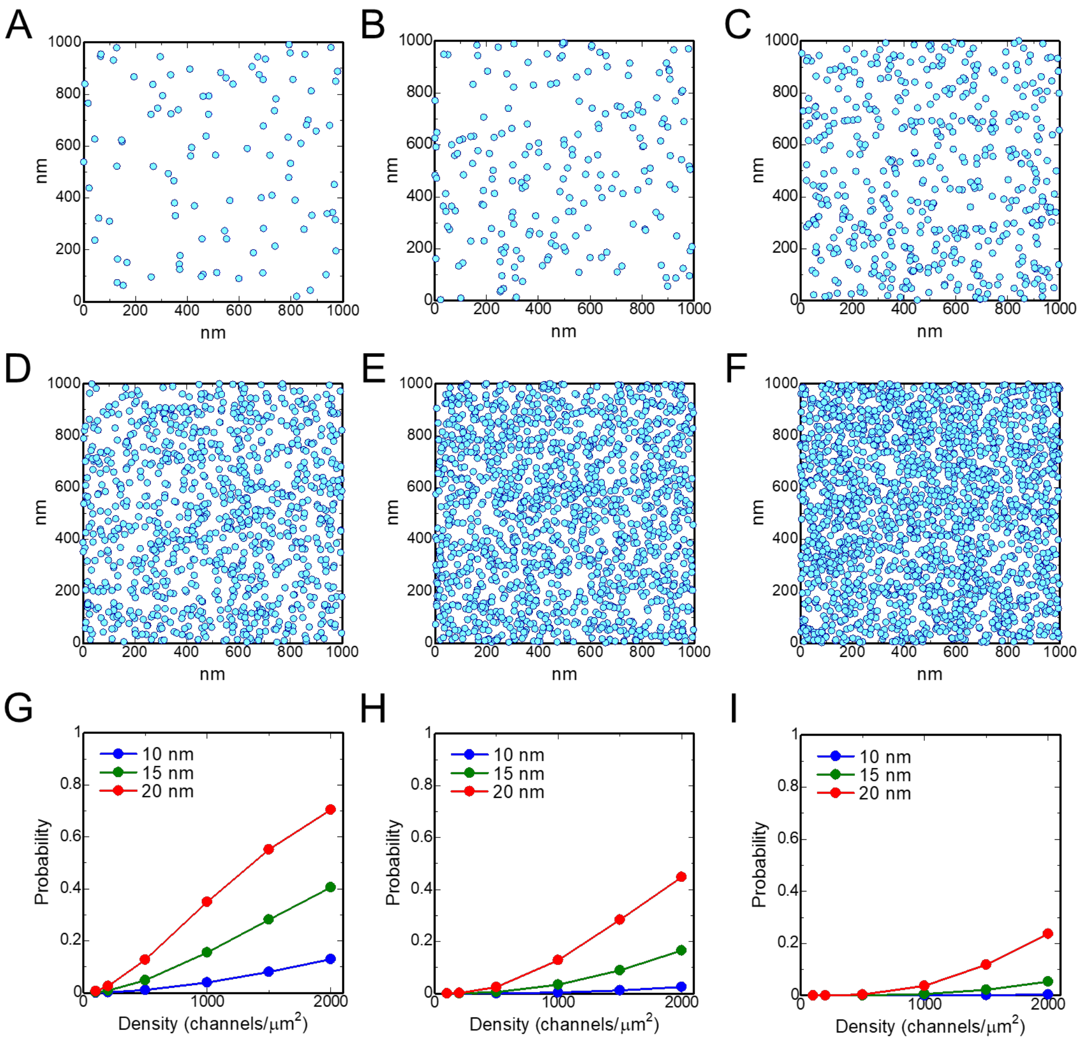

Supplementary Fig.9. Density dependence of the contact probability between particles mimicking resting Na$_v$ channels.

(A-F) Particles 20 nm in diameter were placed randomly in an area of 1 μm^2. The diameter roughly corresponds to the diameter of the resting Na$_v$Ab channel. The center coordinates of the particles were generated randomly without considering the area of the particles, so the particles were allowed to overlap each other. The densities are 100, 200, 500, 1000, 1500, and 2000 channels/μm^2 for A, B, C, D, E and F, respectively. (G, H, I) Contact probability at different densities of particles with diameters of 10, 15 and 20 nm. Probabilities were calculated using 10000 sets of randomly generated snapshots including 100-2000 particles in an area of 1 μm^2. The proportions of particles in contact with at least two (G), three (H) and four (I) other particles were plotted.

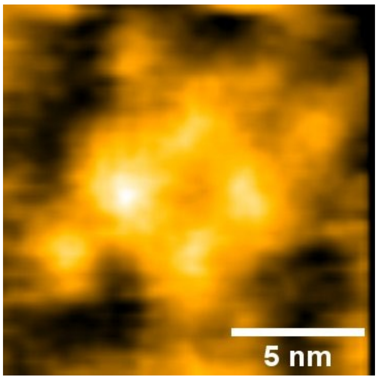

Supplementary Fig.10. E32Q/N49K mutant without a lipid bilayer.

The Na_VAb channel is attached onto Ni^{2+}-coated mica as in the other experiments but coincidentally is outside the lipid bilayer.
