# Supplementary material for: Voltage sensors of a Na+ channel dissociate from the pore domain and form inter-channel dimers in the resting state
Source: Nat Commun. 2023 Dec 19;14:7835. doi: 10.1038/s41467-023-43347-3 (PMC10730821; doi:10.1038/s41467-023-43347-3)
Supplement: Supplementary file 3 — Description of Additional Supplementary Files [file 41467_2023_43347_MOESM3_ESM.pdf]

Description of Additional Supplementary Files

File Name: Supplementary Movie 1
Description: HS-AFM of clustering NavAb (KAV) channels

File Name: Supplementary Movie 2
Description: HS-AFM of deforming tetrameric PDs of the NavAb (KAV) channel

File Name: Supplementary Movie 3
Description: HS-AFM of the NavAb (WT) channel

File Name: Supplementary Movie 4
Description: HS-AFM of the NavAb (N49K) channel

File Name: Supplementary Movie 5
Description: HS-AFM of the NavAb (E32Q/N49K) channel

File Name: Supplementary Movie 6
Description: MD simulation of VSD dimer
